# Surface-Limited Electrodeposition of Continuous Platinum Networks on Highly Ordered Pyrolytic Graphite

**DOI:** 10.3390/nano8090721

**Published:** 2018-09-13

**Authors:** Filippo Farina, Giorgio Ercolano, Sara Cavaliere, Deborah J. Jones, Jacques Rozière

**Affiliations:** Institute Charles Gerhardt Montpellier, UMR CNRS 5253, Aggregates Interfaces and Materials for Energy, University of Montpellier, 34095 Montpellier CEDEX 5, France; filippo.farina@umontpellier.fr (F.F.); giorgio.ercolano@umontpellier.fr (G.E.); deborah.jones@umontpellier.fr (D.J.J.); jacques.roziere@umontpellier.fr (J.R.)

**Keywords:** electrodeposition, platinum, highly oriented pyrolytic graphite, 2D growth, thin films

## Abstract

Continuous thin platinum nanoplatelet networks and thin films were obtained on the flat surface of highly ordered pyrolytic graphite (HOPG) by high overpotential electrodeposition. By increasing the deposition time, the morphology of the Pt deposits can be progressively tuned from isolated nanoplatelets, interconnected nanostructures, and thin large flat islands. The deposition is surface-limited and the thickness of the deposits, equivalent to 5 to 12 Pt monolayers, is not time dependent. The presence of Pt (111) facets is confirmed by High Resolution Transmission Electron Microscopy (HRTEM) and evidence for the early formation of a platinum monolayer is provided by Scanning Transmission Electron Microscopy and Energy Dispersive X-rays Spectroscopy (STEM-EDX) and X-ray Photoelectron Spectroscopy (XPS) analysis. The electroactivity towards the oxygen reduction reaction of the 2D deposits is also assessed, demonstrating their great potential in energy conversion devices where ultra-low loading of Pt via extended surfaces is a reliable strategy.

## 1. Introduction

Carbon-supported platinum electrocatalysts are employed in the electrodes of Proton Exchange Membrane Fuel Cells (PEMFCs), but due to the scarcity of platinum and its cost [1], efforts to reduce the noble metal loading have driven research towards the development of strategies maximising its utilisation [2], including metal@platinum core@shell nanoparticles [3], platinum nanostructures [4] and thin films [5]. Several methods such as Atomic Layer Deposition (ALD) [6,7], Pulsed Laser Deposition (PLD) [8], Surface-Limited Redox Replacement (SLRR) [9], and Magnetron Sputtering (MS) [10] allow the preparation of extended Pt surfaces on various supports including carbon [11].

Electrodeposition of Pt in galvanostatic and potentiostatic modes is conventionally characterized by the 3D growth of platinum, which forms spherical, or flower-like, agglomerates [12,13,14]. Achieving the formation of thin Pt structures via electrodeposition therefore represents a real challenge. We recently observed that pulsed electrodeposition at high overpotentials can produce crystalline nanoplatelets on carbonaceous surfaces. Thin 2D Pt nanoplatelets were grown on electrospun carbon fibres and on nanotubes developed from their surfaces, and the resulting materials were evaluated as electrocatalysts for the Oxygen Reduction Reaction (ORR) in the cathode of a PEMFC, demonstrating enhanced exploitation of the noble metal [15,16]. Such nanofibres and nanotubes do not lend themselves to in-depth investigations using advanced microscopies, and so to further our understanding of this high overpotential electrodeposition method we turned our attention to the model surface of Highly Ordered Pyrolytic Graphite (HOPG), and herein describe our findings. The aim is to investigate the process occurring on a flat carbon surface at the nanoscale, leading to the formation of crystalline nanoplatelets, interconnected nanostructures, and thin films. To achieve deposition with a single nucleation step, followed by increasing Pt growth for better insights into the deposit formation process, the electrodeposition approach developed in our previous work was modified to consider only a single potential pulse of varying duration. The choice of HOPG as a model surface is due to its relative flatness as well as to its structural similarity to the graphitized regions of the surface of the carbon nanofibers and nanotubes that inspired this work [15,16]. Furthermore, the possibility of exfoliating HOPG to obtain graphene layers [17], which can be further catalysed with platinum, will open novel perspectives of applications.

While studies of platinum electrodeposition on various carbonaceous surfaces have been reported [18,19,20], it appears that the process has been investigated mainly for slight overpotential conditions and not at the very high overpotentials (i.e. in the region of strong H_2_ evolution) that are the conditions chosen here. It was previously reported that interconnected Pt structures can be obtained by applying an overpotential of −500 mV vs. SCE (−259 SHE) [21,22]. Such a high overpotential could in principle force two-dimensional growth in through underpotential deposition of hydrogen (H_upd_) on the Pt surface, which prevents or impedes diffusion of the Pt precursor towards the surface of the newly electrodeposited Pt layers [23]. Spontaneous deposition of Pt agglomerates, nanoparticles, and networks on HOPG was induced from water as well as non-aqueous polar solvents [24,25,26] and was explained in terms of the reduction of Pt by oxidized defect sites on the surface and step-edges [24]. However, the mechanism appears to be more complex, involving not only graphite step-edges, but also hydrogenated sites [27,28]. As a consequence, Pt nuclei may be already present before the application of the overpotential to the electrode [28].

In this paper, we investigate the effect of a high overpotential single-pulse length on the morphology and crystal structure of the Pt deposits on the flat model carbon surface HOPG for better insights on the deposition mechanism and 2D growth of extended metal surfaces using microscopy and surface analysis techniques.

## 2. Materials and Methods 

Highly Oriented Pyrolytic Graphite (HOPG, ZYH grade, 12 mm × 12 mm from Veeco-Bruker, Camarillo, CA, USA) was used as the working electrode in a two-electrode cell, and a 5 cm × 5 cm graphite sheet (Goodfellow) was used as the counter-shorted reference electrode (RE/CE). The working electrode assembly was masked with Kapton^®^ tape (RS Components SAS, Beauvais, France), leaving a 6 mm × 6 mm HOPG surface exposed to the precursor solution, and the HOPG back-plate was connected with a platinum wire to the potentiostat (Bio-Logic^®^ SP300, Bio-Logic SAS, Seyssinet-Pariset, France). Electrodeposition experiments were performed at −3 V vs. RE/CE (measured to be equivalent in the same cell geometry to −1.9 V vs. Ag/AgCl) for various duration times (from 5 to 200 s), followed by 60 s at open circuit voltage (OCV). The deposition solution was a solution of 3 mM hexachloroplatinic acid hexahydrate (≥99.9% trace metal basis) and 0.5 M sodium chloride in MilliQ water (18 MΩ cm). All chemicals were provided by Sigma-Aldrich, St. Louis, MO, USA. An iR correction was performed before each electrodeposition step with the typical electrical resistance being in the range 10–20 Ω; the distance between the electrodes was fixed at 5 cm. Before electrodeposition, the solution was cooled to ~4 °C in an ice bath and purged with nitrogen to remove the dissolved oxygen. During the deposition, the precursor solution was stirred at 900 rpm to remove the hydrogen bubbles that evolve on the surface of the HOPG and to limit the thickness of the precursor diffusion layer. Prior to any characterization, the deposited sample was thoroughly rinsed with MilliQ grade water. Tapping Mode Atomic Force Microscopy (TM-AFM) images were acquired with a Bruker Nanoman (Bruker SAS, Palaiseau, France) driven by Nanoscope 5 electronics. The cantilever tips were Silicon Point Probe Plus^®^ NCSTR (Nanosensors, Neuchâtel, Switzerland; force constant 6.5 N/m, resonance frequency 157 kHz). All the image treatments and the thickness measurements were performed using WSxM 4.0 Beta 8.2 [29] and the resulting data were treated with the Fityk software [30] to correct the background. Particle size measurements and Fast Fourier Transforms were performed with ImageJ 1.48 v (U. S. National Institutes of Health, Bethesda, MD, USA). High Resolution Transmission Electron Microscopy (HRTEM), Scanning Transmission Electron Microscopy (STEM) micrographs and Energy Dispersive X-rays Spectroscopy (EDX) data were obtained with a JEOL 2200FS (Source: FEG) microscope (JEOL Europe SAS, Croissy-sur-Seine, France) operating at 200 kV and equipped with a CCD camera Gatan USC (16 MP; Gatan, Evry, France). The samples were prepared by carefully peeling away flakes of HOPG and depositing them onto a Cu grid with a drop of silver paste. Scanning electron micrographs were acquired by Field Emission-Scanning Electron Microscopy (FE-SEM) using a Hitachi S-4800 microscope (Hitachi Europe SAS, Velizy, France). The surface composition of the samples was investigated by X-ray Photoelectron Spectroscopy (XPS) on an ESCALAB 250 (Thermo Electron, Villebon Sur Yvette, France). The X-ray excitation was provided by a monochromatic Al K_α_ (1486.6 eV) source, and the analysed surface area was 400 µm^2^. A constant analyser energy mode was used for the electron detection (20 eV pass energy). Detection of the emitted photoelectrons was performed perpendicular to the surface sample. Data quantification was performed on the Avantage software (Thermo Fisher Scientific, Waltham, MA, USA), removing the background signal using the Shirley method. The surface atomic concentrations were determined from photoelectron peaks areas using the atomic sensitivity factors reported by Scofield. Binding energies of all core levels are referenced to the C–C bond of C 1s (284.8 eV). Linear sweep voltammetry (LSV) was performed at 20 mV s^−1^ in O_2_ saturated 0.1 M HClO_4_ on a Bio-Logic^®^ SP300 potentiostat (see above); the working electrode was an HOPG-Pt sample connected to the potentiostat with a Pt wire, while the counter electrode was a graphite rod (Alfa Aesar, Heysam, UK) and reference used was an Ag/AgCl electrode (Fisher Scientific, Illkirch, France; E = 0.197 V vs. NHE). The potentials in the manuscript were reported vs. NHE.

## 3. Results and Discussion

### 3.1. TM-AFM Analysis

**Figure 1:**
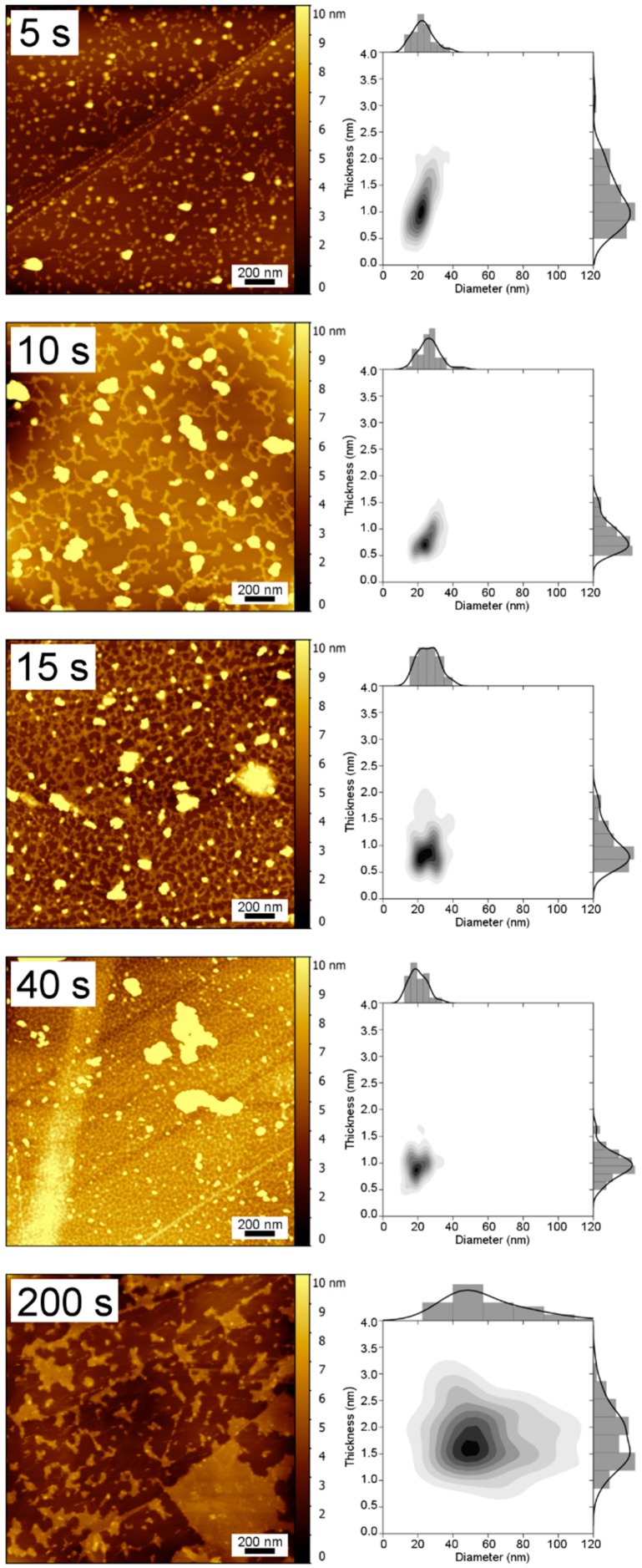
TM-AFM images of Pt on HOPG electrodeposited at different pulse times (**left**, scale bars are 200 nm) with the respective thickness and diameter bivariate distributions (**right**).

Tapping Mode Atomic Force Microscopy (TM-AFM) images of electrodeposited Pt samples prepared with different pulse durations are shown in Figure 1, together with their thickness and diameter distributions. 

Only separate Pt nanoplatelets were visible for a 5 s deposition time, while thin Pt networks started to clearly form after a 10 s deposition, and progressively covered the surface of the HOPG substrate up to 40 s and, at 200 s, thin large flat Pt islands were fully formed. Larger agglomerates of Pt were also present along all the samples, whatever the pulse duration, possibly formed either during the open circuit voltage (OCV) step of the electrodeposition process or as a result of H_2_-induced precipitation [23]. The Pt morphologies resulting from the application of this overpotential method in the range 5–40 s appear to be partially similar to previous reports, but they clearly show that the substrate surface is progressive covered with a network of interconnected flat Pt nanostructures of low thickness [21,22]. An increase of the degree of interconnection of the Pt nanoplatelets is clearly visible with an increase in deposition time. A general trend can be derived for the sample series, however one might take into account a certain intra-sample inhomogeneity.

**Figure 2:**
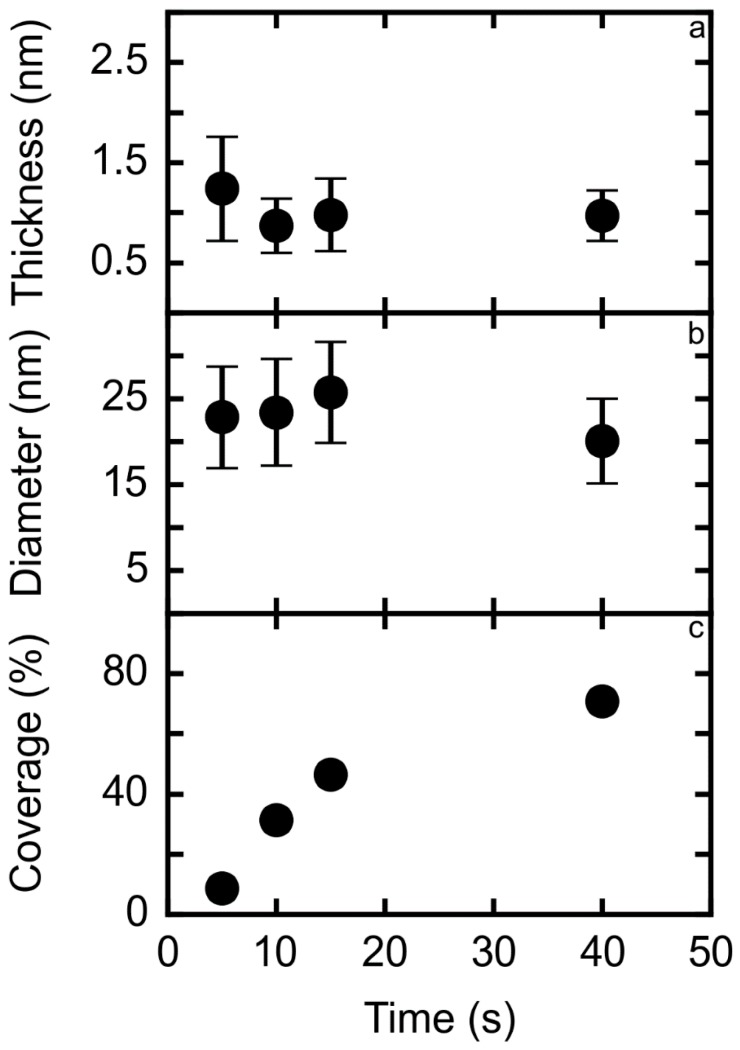
Dependence of average Pt nanoplatelets thickness (with standard deviation) (**a**) average Pt nanoplatelets diameter (with standard deviation). (**b**) and percentage of covered surface. (**c**) on the deposition time. The sample obtained with a 200 s pulse is not included for clarity (see Table 1).

As shown in Figure 2a and Table 1, the thickness of the Pt nanoplatelets only slightly changed with deposition time. Most of the nanoplatelets did not grow over 4 nm in thickness even at longer deposition times (Table 1), with the average being comprised between 1.0 and 2.5 nm (equivalent to 5 to 12 Pt monolayers). Pt deposits with comparable thickness and diameter were reported for slight overpotential conditions and shorter times as well, but there was no evidence of interconnected or extended surface structures [31].

Similarly, the average nanoplatelet diameter did not vary greatly with the deposition time (Figure 2b) and the size distribution is reasonably similar for all the samples (Figure 1). Although the diameter of the platinum islands is comparable to some reported for other overpotential conditions [32], the present high overpotential conditions lead to a distinct geometry in that the diameter is roughly 20 times the thickness for samples in the 5–40 s time interval, confirming the presence of flat nanoplatelets and thus two-dimensional growth.

The percentage of surface coverage increases with the deposition time (Figure 2c), indicating a surface-limited electrodeposition in the range 5–40 s. The sample prepared with 200 s deposition duration presented different values both for size distribution and dimension ratio due to its clear film-like morphology, dissimilar to the interconnected networks observed at shorter deposition times. It should be noticed that the obtained morphologies and trends are reproducible, while the current densities obtained for Pt electrodeposition can be slightly different from one sample to another. This is due to the different conditions of the HOPG surface exposed by cleavage of the C–C stacking [28].

Different overpotential conditions may affect the aspect of the resulting Pt deposit, and phenomena such as secondary nucleation, surface diffusion, and coalescence should be taken into account to explain the formation of the different structures and morphologies [32]. The supporting electrolyte also takes part in the Pt growth mechanism and in determining the final morphology of the resulting deposit [22,33], its role depending on the concentration, and on the applied potential. In this work, a chloride supporting electrolyte was chosen for compatibility with the Pt precursor (H_2_PtCl_6_) possibly releasing the same anion. The 0.5 M NaCl solution provided a high concentration of Cl^−^ anions that may adsorb on the surface of the newly formed Pt structures, preventing further Pt atoms from depositing and growing in a three dimensional way [21,33]. Simultaneous adsorption of H and Cl species on Pt could also occur, possibly synergistically [34,35], or competitively, with the possibility also that at high overpotential only protons are adsorbed [36]. Adsorbed anions may also be partially responsible for the smaller Pt particles migrating from one active site to another or to other Pt surfaces [22,37]. This phenomenon may represent a possible or at least partial explanation for the presence of large Pt agglomerates in these samples. In the very high overpotential conditions used in this work, it is likely that no Cl^−^ is adsorbed on the Pt surfaces, due to the strong evolution of gaseous H_2_. The substrate also plays a role in Pt growth. Indeed, spontaneous electroless deposition on HOPG has been observed from Pt solutions, indicating the presence of metal seeds before the overpotential electrodeposition. The reducing agents for this spontaneous deposition are likely HOPG step edges that get oxidized in solution [28].

### 3.2. Electron Microscopy Analysis

**Figure 3:**
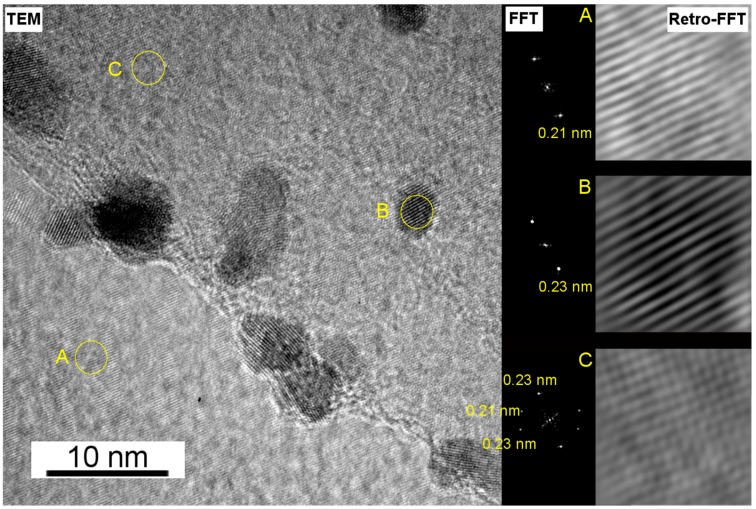
HRTEM micrograph of a slice of HOPG decorated with Pt nanoplatelets (**left**). FFT images of the three selected areas (**centre**) and the derived inverse-FFT (**right**).

High resolution transmission electron microscopy (HRTEM) micrographs of a Pt on an HOPG sample electrodeposited using a 15 s pulse were acquired to confirm the chemical nature and morphology of the deposit (Figure 3). The dimensions of the nanoplatelets were around 10 nm. The Fast Fourier Transform (FFT) analysis on selected areas of the TEM image, shown in Figure 3, presents three regions denoted A, B, and C with different d-spacings for the regions where carbon (A) and platinum (B) were clearly distinguishable.

**Figure 4:**
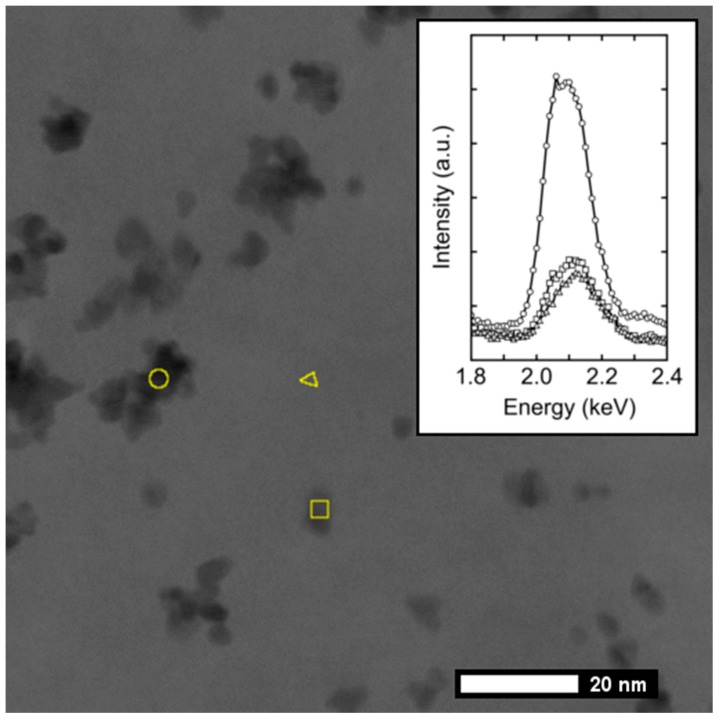
STEM micrograph of a flake of HOPG decorated with Pt aggregates (○) and Pt nanoplatelets (□); the comparison of their EDX spectra with the apparent bare surface of HOPG (∆) is reported in the inset.

In region A, only a single maximum at 0.21 nm is observed, comparable with the d-spacing of the (011) plane of graphite (0.2031 nm in JCPDS 96-901-2231). Region B clearly corresponds to a Pt platelet with a marked contrast over the carbon background. Indeed, in this region, a d-spacing of 0.23 nm was measured, fully compatible with that of the (111) facet of Pt (0.2299 nm in JCPDS 96-151-2257). In region C, no clear contrast nor evidence for platinum is visible from the TEM image. However, the FFT displays 3 maxima (one of 0.21 nm and two of 0.23 nm) demonstrating the presence of an extremely thin platinum layer, which was further corroborated by use of other techniques. Thus, to seek further evidence for the presence of Pt layers in areas where no obvious contrast was visible by imaging only, Energy Dispersive X-ray Spectroscopy (EDX) spectra on a Scanning Transmission Electron Microscopy (STEM) micrograph were recorded. Similarly to a previous report [38], local elemental analysis was performed on three different morphological features (Pt aggregate, Pt nanoplatelet and apparently bare HOPG) situated in another area of the same sample of Figure 3. The EDX spectra of the three zones highlighted in Figure 4 unquestionably show a Pt signal (Figure 4, inset) also in areas where no Pt nanoplatelets are visible by STEM. To conclude, the electron microscopy results depicted in Figure 3 and Figure 4 provide complementary evidence for the presence of electrodeposited Pt all over the HOPG surface, demonstrating the early stage formation of a continuous ultra-thin electrodeposited Pt layer.

### 3.3. XPS Characterization

**Figure 5:**
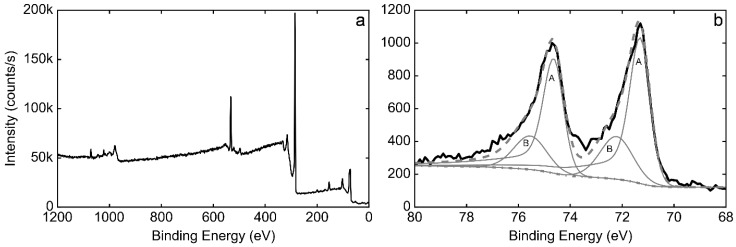
Survey XPS spectrum of a HOPG-Pt sample deposited for 200 s (**a**) and high-resolution spectrum of the Pt 4f region with peaks deconvolution (**b**).

Surface analysis by X-ray Photoelectron Spectroscopy (XPS) was performed on a sample electrodeposited for 200 s both to further endorse the deposition of platinum, as well as to determine its oxidation state (Figure 5). The A bands of the Pt doublet observed on the sample (Pt 4f_7/2_ at 71.3 eV, Pt 4f_5/2_ at 74.6 eV) correspond to metallic Pt deposited on the HOPG surface. Additional contributions of B bands (72.2 and 75.5 eV respectively) were detected and related to the presence of oxidized forms of Pt, such as PtO_ads_ and/or Pt(OH)_2_ [39]. The deconvolution of the Pt 4f doublet peaks indicates that Pt^0^ is the majority species (73.6% of the total signal, comparable to Pt freshly electrodeposited onto graphite substrates previously reported [40]), rather than ionic Pt, which confirms the predominant metallic nature of the deposit and thus the suitability of the technique to prepare catalytically active Pt thin films.

The resulting Pt 4f spectrum is consistent with those reported for Pt films on HOPG with different thicknesses prepared by sputtering [41,42]. It is known that a Pt film thicker than 0.1 Å can already produce an XPS profile with two differentiated peaks, and since the sample prepared in this work also presented some Pt aggregates, the spectrum is the result of the presence of both 2D and 3D structures. 

### 3.4. Electrocatalytic Activity

**Figure 6:**
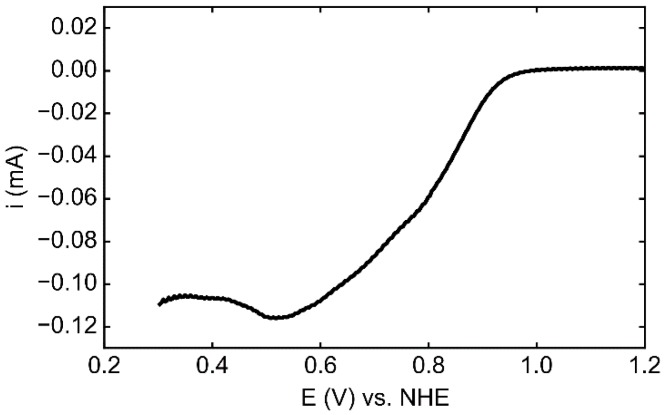
LSV of a HOPG-Pt sample electrodeposited for 200 s in O_2_ saturated 0.1 M HClO_4_ at 20 mV s^−1^.

To assess the catalytic properties of the electrodeposited metal towards the oxygen reduction reaction (ORR), an HOPG-Pt sample deposited for 200 s was analyzed in O_2_ saturated 0.1 M HClO_4_ in a classical three-electrode configuration (scan from 1.2 to 0.3 V vs. NHE). The linear sweep voltammetry (LSV) shown in Figure 6 demonstrates that the deposited Pt^0^ is active, with an onset potential of ca 0.98 V vs. NHE. No reliable mass activity values could be derived due to the experimental conditions that are far from steady-state.

## 4. Conclusions

In conclusion, Pt nanoplatelet networks and Pt thin films were deposited on HOPG by high overpotential electrodeposition with a morphology that depends on pulse duration. The Pt networks formed using 5–40 s pulses comprised of extended islands of average thickness of 1 nm that were fairly homogeneous in diameter (20 times the thickness). Ultra-thin (<2 nm) Pt films are formed at a longer pulse time, 200 s, in which the exposed surfaces are Pt (111) facets. HR-TEM and XPS analysis confirm the metallic nature of the platinum deposit. These greater insights on the morphology of Pt structures deposited at high overpotential could only be obtained on a model carbon surface such as HOPG and will now serve to achieve complete formation of conformal and continuous ultra-thin Pt films on other carbonaceous surfaces (e.g., nanofibers, nanotubes, etc…) of applicability in energy conversion, such as the development of ultra-low platinum loaded fuel cell electrodes.

## Figures and Tables

**Table 1:** Dependence of average thickness and diameter of the electrodeposited Pt nanoplatelets on the deposition time. Surface coverage percentage was calculated on the AFM micrographs shown in Figure 1.

Sample	Thickness (nm)	Diameter (nm)	Surface Coverage (%)
5	1.24 ± 0.52	22.8 ± 6	8.7
10	0.87 ± 0.27	23.4 ± 6	31.4
15	0.98 ± 0.36	25.8 ± 6	46.5
40	0.97 ± 0.25	20.1 ± 5	70.7
200	1.82 ± 0.52	72.0 ± 69	44.9
